# Chi-miR-30b-5p inhibits dermal papilla cells proliferation by targeting *CaMKIIδ* gene in cashmere goat

**DOI:** 10.1186/s12864-020-06799-1

**Published:** 2020-06-26

**Authors:** Yuelang Zhang, Sizhe Xia, Tianci Wang, Shanhe Wang, Dan Yuan, Fang Li, Xin Wang

**Affiliations:** 1 Key Laboratory of Animal Genetics, Breeding and Reproduction of Shaanxi Province, College of Animal Science and Technology, Northwest A&F University, Yangling, 712100 Shaanxi China; 2 Present address: College of Animal Science & Technology, Northwest A&F University, No.3 Taicheng Road, Yangling, 712100 Shaanxi China; 3 The College of Life Science, Northwest University, Xi’an, 710069 Shaanxi China

**Keywords:** Cashmere goat, miRNA-seq, Chi-miR-30b-5p, DP cells, Proliferation

## Abstract

**Background:**

During goat embryonic morphogenesis and postnatal initiation of hair follicle (HF) regeneration, dermal papilla (DP) cells play a vital role in hair formation. Growing evidence shows that microRNAs (miRNAs) participate in HF development and DP cell proliferation. However, the molecular mechanisms have not been thoroughly investigated.

**Result:**

In this study, we utilized miRNA sequencing (miRNA-Seq) to identify differentially expressed miRNAs at different HF cycling stages (anagen and telogen). MiRNA-Seq has identified 411 annotated miRNAs and 130 novel miRNAs in which 29 miRNAs were up-regulated and 32 miRNAs were down-regulated in the anagen phase compared to the telogen phase. Target gene prediction and functional enrichment analysis indicated some major biological pathways related to hair cycling, such as Wnt signaling pathways, ECM-receptor interaction, VEGF signaling pathway, biosynthesis of amino acids, metabolic pathways, ribosome and oxidative phosphorylation. Also, we explored the function of chi-miR-30b-5p in regulating hair growth cycle. Similar to the HF cycling, DP cells were isolated from skin and used to investigate miRNA functions. The MTT and EdU assays showed that the viability and proliferation of DP cells were inhibited or promoted after the transfection of chi-miR-30b-5p mimic or inhibitor, respectively. Bioinformatics analysis revealed *CaMKIIδ* as a candidate target gene of chi-miR-30b-5p, and the dual-luciferase and western blot assay demonstrated that chi-miR-30b-5p bound to the 3’UTR of *CaMKIIδ* and further inhibited its translation.

**Conclusion:**

Chi-miR-30b-5p was found to be highly expressed in the telogen than that in the anagen phase and could inhibit the proliferation of DP cells by targeting *CaMKIIδ*. Our study provides new information on the regulatory functions of miRNAs during HF development.

## Background

Cashmere is a vital raw material for the clothing industry with the characteristics of great resilience, high moisture absorption, and fine heat preservation [1]. The Shanbei white cashmere goat is noted for its remarkable and fine fiber production features. During postnatal life, HF undergoes three periods of alternation: anagen (active growth), catagen (regression), and telogen (relative resting) [2]. The transformation and growth of HF are regulated by various complex factors in the skin [3, 4]. Vitamin D receptor shows an essential role in anagen-catagen transformation [5]. Some anagen inducing signals are identified by mutant mouse experiments, like Wnt/β-catenin, BMP antagonist (Noggin), and Shh [6, 7]. Furthermore, FGF5 shows an important role in the occurrence of catagen [8]. A new hair germ generates after the telogen-to-anagen transition, and the transition between these two periods is crucial for hair regeneration. Therefore, the two stages are selected for miRNA-seq in this study.

MiRNAs are a class of small endogenous non-coding RNAs, roughly 18 ~ 25 nt in length. They can post-transcriptionally regulate gene expression by binding to the seed region (the 5′ end sequences of miRNA) and the cognate 3’UTR of mRNAs sequences to inhibit translation or induce mRNA decay [9, 10]. Many studies have indicated that miRNAs play crucial roles in skin development and regeneration [11–13]. It has identified and characterized miR-214, miR-21, miR-24 and miR-200 family in HF biology of human or mouse [14–17]. MiR-214 inhibits the expression of *ctnnb1* (a key factor of Wnt signaling pathway) to regulate HF cycling and skin morphogenesis [14]. MiR-24 overexpression mice display the altered HF structure and a marked defect with thinner hair cover through the repression of *Tcf*-3 (hair keratinocyte stemness regulator) [15]. MiR-21 promotes cell proliferation and migration by preventing the inhibitory effects of *BMP4* in primary keratinocyte cells [16]. MiR-200 families regulate the orientation in hair germ and cell adhesion, leading to precise hair morphogenesis and cell fate specification [17]. Previous study has shown that miR-30b-5p performs its biological function as a tumor inhibitor in human hepatocellular carcinoma [18]. In gastric cancer, miR-30b-5p suppresses tumor growth and promotes apoptosis [19]. Also, miR-30b-5p suppresses tumor proliferation in non-small cell lung and colorectal cancer [20]. Yuan et al. observed that chi-miR-30b-5p expressed significantly lower in the anagen than that in the telogen of cashmere goat [21]. However, whether chi-miR-30b-5p plays a similar function in HF remains unknown.

In order to explore the role of miRNAs in cashmere development and cycling, the differentially expressed miRNAs between the telogen and anagen were identified using miRNA-seq. Then we further examined the function of chi-miR-30b-5p in regulating hair cycle and DP cells proliferation, which would help to illustrate the regulation mechanism of miRNA on hair cycle.

## Results

### Sequencing of miRNAs

**Fig. 1:**
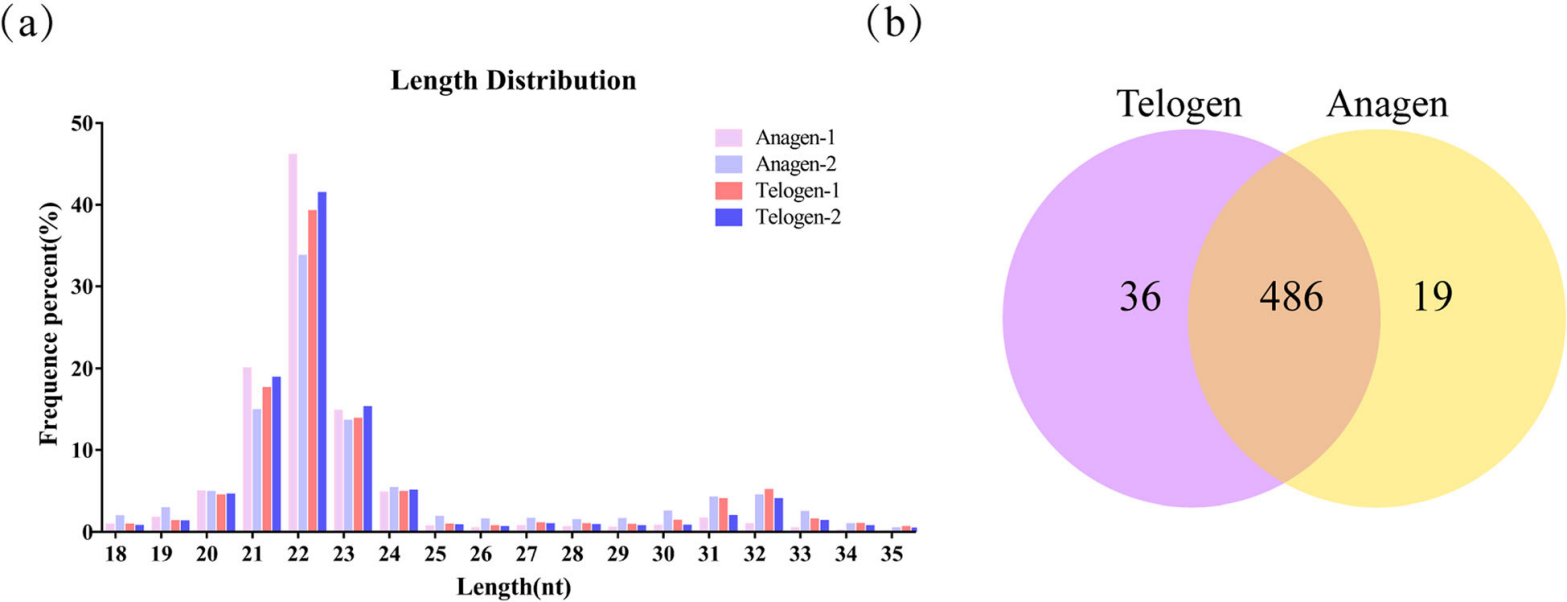
Sequence length distribution and differential miRNAs analysis. (a) Sequence length distribution of the filtered clean reads. (b) Venn diagram of specifically expressed miRNAs at the two stages of HF cycling in cashmere goats

Two small RNA (sRNA) libraries were constructed from the anagen and telogen phases respectively to identify the miRNAs involving cashmere HF cycle. MiRNA seq data showed the average of 13,503,395 clean reads were retained from raw data (Table 1). Generally speaking, the length interval of mammal sRNA is 18 ~ 35 nt. Therefore, the clean reads within the range were screened for subsequent analysis (Additional file 1). To evaluate the sequencing quality, the length distribution was aligned out from the four libraries (Fig. 1a). Most of the sequences were arranged between 18 ~ 25 nt, and the length of 22 nt, which is the typical length of miRNAs, had the highest proportion.

**Table 1:** Summary of reads mapping to the reference genome

Type	Anagen-1	Anagen-2	Telogen-1	Telogen-2	Total
Raw reads	11,266,194	11,520,277	16,749,840	15,356,591	54,892,902
N% > 10%	117	225	61	14	131
low quality	28,575	27,106	39,280	36,885	28,575
5’adapter contaminant	206	550	686	437	1879
3 adapter null or insert null	114,188	142,714	260,037	195,312	309,500
with ployA/T/G/C	4883	9450	10,050	8548	32,931
Clean reads	11,118,225	11,340,232	16,439,726	15,115,395	54,013,578

In order to analyze the expression and distribution of miRNAs, all of the filtered clean reads were mapped with the goat genome sequence. Totally 25,870,562 (50.08%) reads were aligned to the same strand in the reference sequence direction and 16,148,170 (31.26%) reads were mapped to the antisense strand (Additional file 1).

### Expression analysis of annotated and novel miRNAs

First, the clean reads from above were mapped to the annotated miRNA sequence of *Capra hircus* in miRbase 20.0. A total of 21,154,428 reads were mapped to the annotated miRNA, which represented 50.35% of the total mapped reads (Additional file 2). As a result, 411 annotated mature miRNAs and 130 novel miRNAs were determined at the two stages of HFs cycle. At the same time, 259 annotated miRNA and 139 novel miRNA precursors were identified (Additional files 3 and 4).

The expression levels of novel and annotated miRNAs in each library were counted and normalized by TPM (Additional file 5). A total of 486 miRNAs were co-expressed at the two HF cycling stages, whereas 36 and 19 miRNAs were specifically expressed in telogen and anagen, respectively (Fig. 1b).

**Fig. 2:**
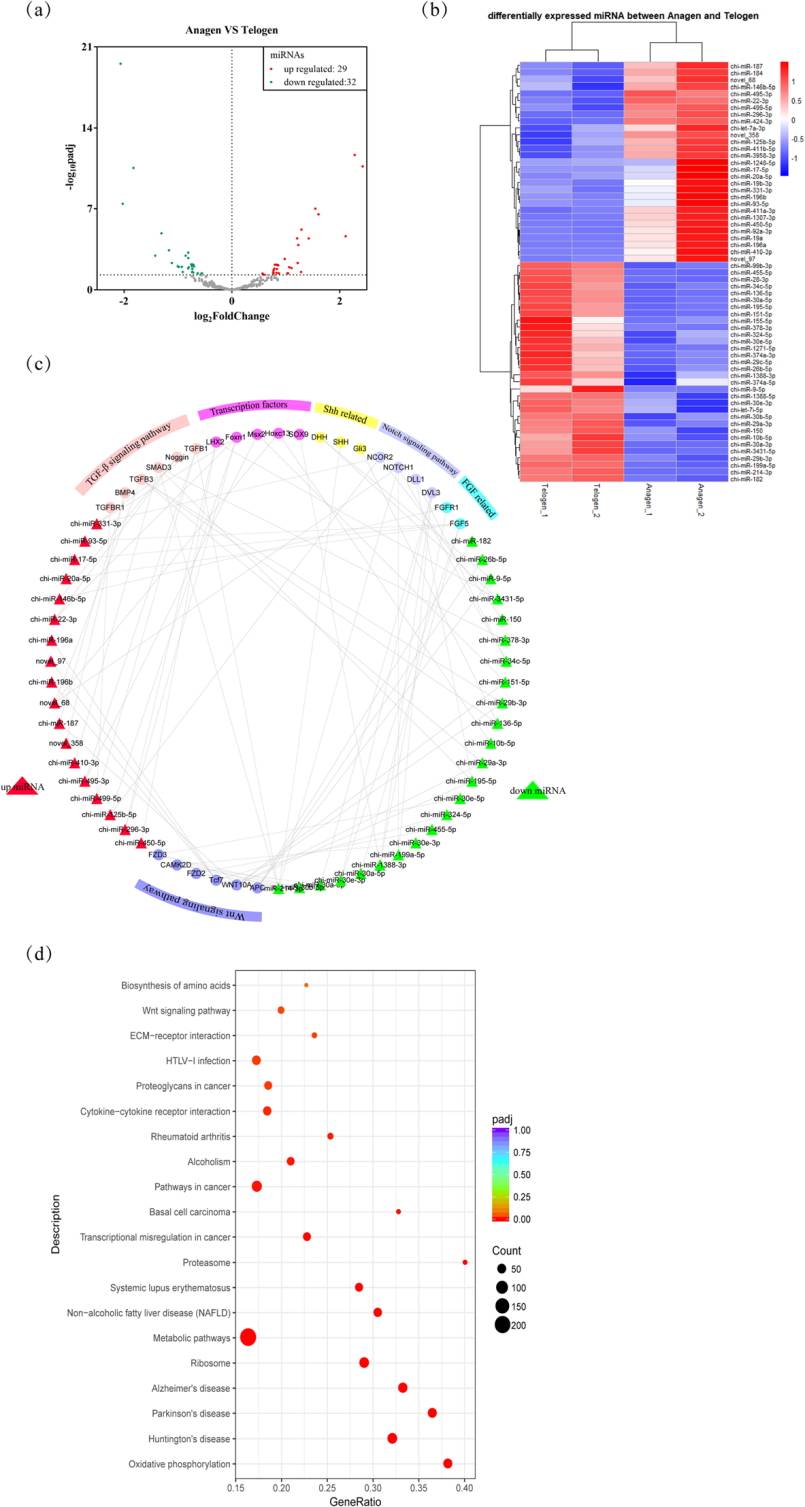
Differentially expressed miRNAs in goat skin between anagen and telogen of HF cycle (P-adjust value ≤0.05). (a) Volcano map of differentially expressed miRNAs, green dot, miRNAs down-regulation; red dot, miRNAs up-regulation; (b) Heat map clustering of differentially expressed miRNAs, Log10 (TPM + 1) value is used for clustering. Red represents high-expression miRNA, while blue represents low-expression miRNA. (c) Network of differentially expressed miRNAs and their potential targets genes involving hair follicle development and cycle. (d) The top 20 KEGG pathways of target genes of differentially expressed miRNAs in goat skin between anagen and telogen. GeneRatio indicates the ratio of target genes enriched in the pathway among genes annotated in the pathway

Based on negative binomial distribution, the differentially expressed miRNAs between the two cycling stages were analyzed, and p-adjust value < 0.05 was considered as significantly differentially expressed miRNAs. 58 annotated and 3 novel differentially expressed miRNAs were identified between the telogen and anagen. Compared with the telogen, 29 miRNAs were up-regulated and 32 miRNAs were down-regulated in the anagen (Fig. 2a, Additional file 6). The differentially expressed miRNAs in each library were clustered and visually viewed in a heat map (Fig. 2b).

### MiRNA targets prediction and KEGG pathway analysis

Target genes of differentially expressed miRNAs were predicted by miRanda and TargetScan. The important genes involving the processes of HF cycle and development were identified. Figure 2c showed the regulatory relationships between miRNAs and genes.

Furthermore, the target genes were conjointly analyzed with the differentially expressed genes (DEGs) (q value ≤0.05) in the anagen and telogen in our previous study [22], resulting in 2347 identified target genes. KEGG (Kyoto encyclopedia of genes and genomes) pathway analysis showed that the 2347 target genes were annotated for 289 biological functions. Some traditional pathways associated with HF cycling were presented in the top 20 KEGG pathways, including ECM-receptor interaction and Wnt signaling pathway (Fig. 2d, Additional file 7). In addition, the results indicated that biosynthesis of amino acids, metabolic pathways, ribosome, and oxidative phosphorylation played an important role in HFs cycling.

### Chi-miR-30b-5p suppressed the proliferation of DP cells

**Fig. 3:**
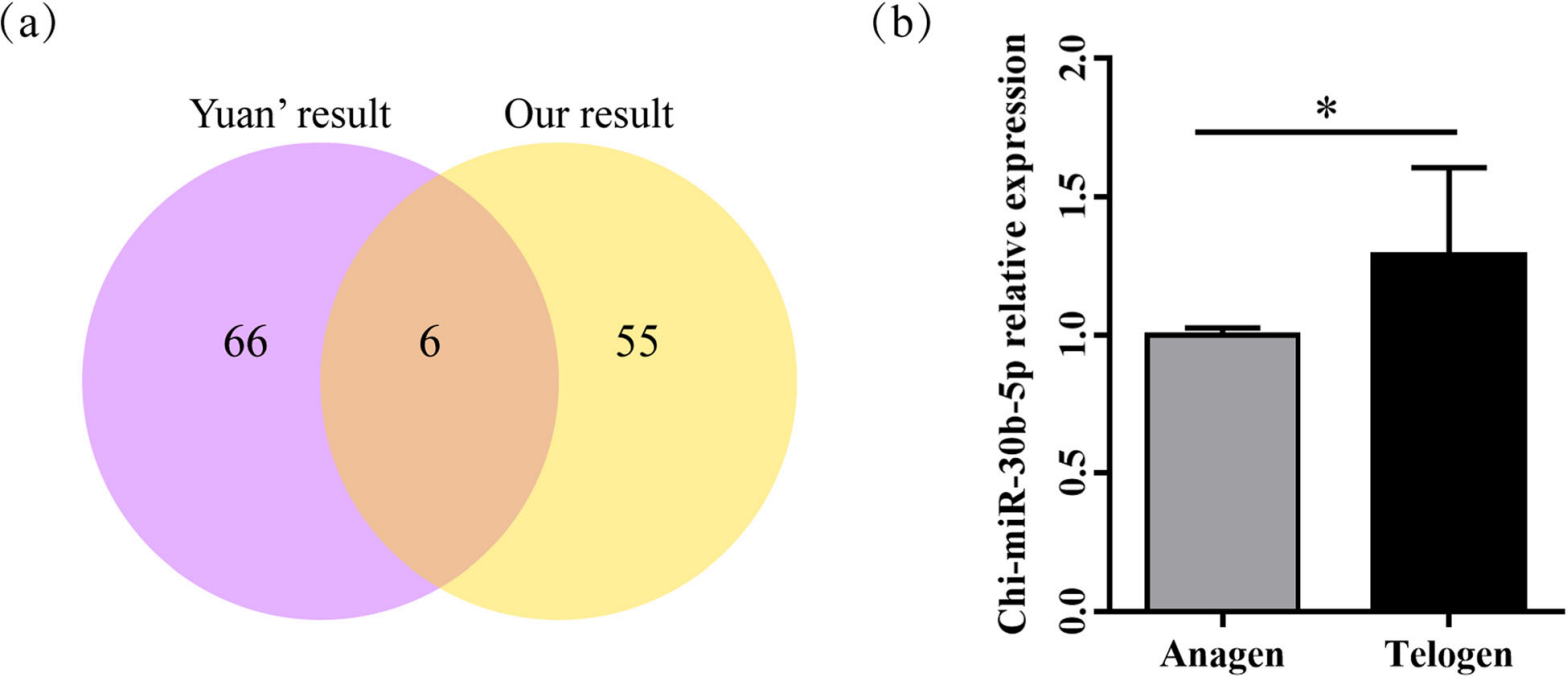
Chi-miR-30b-5p screening and validating by RT-qPCR. (a) Venn diagram of specifically differentially expressed miRNAs in our sequencing result and Yuan’s. (b) RT-qPCR of chi-miR-30b-p expression in goat skin between anagen and telogen. The expression of chi-miR-30b-5p were normalized to U6. Values are mean ± SEM for 3 biological replicates, *P < 0.05

**Fig. 4:**
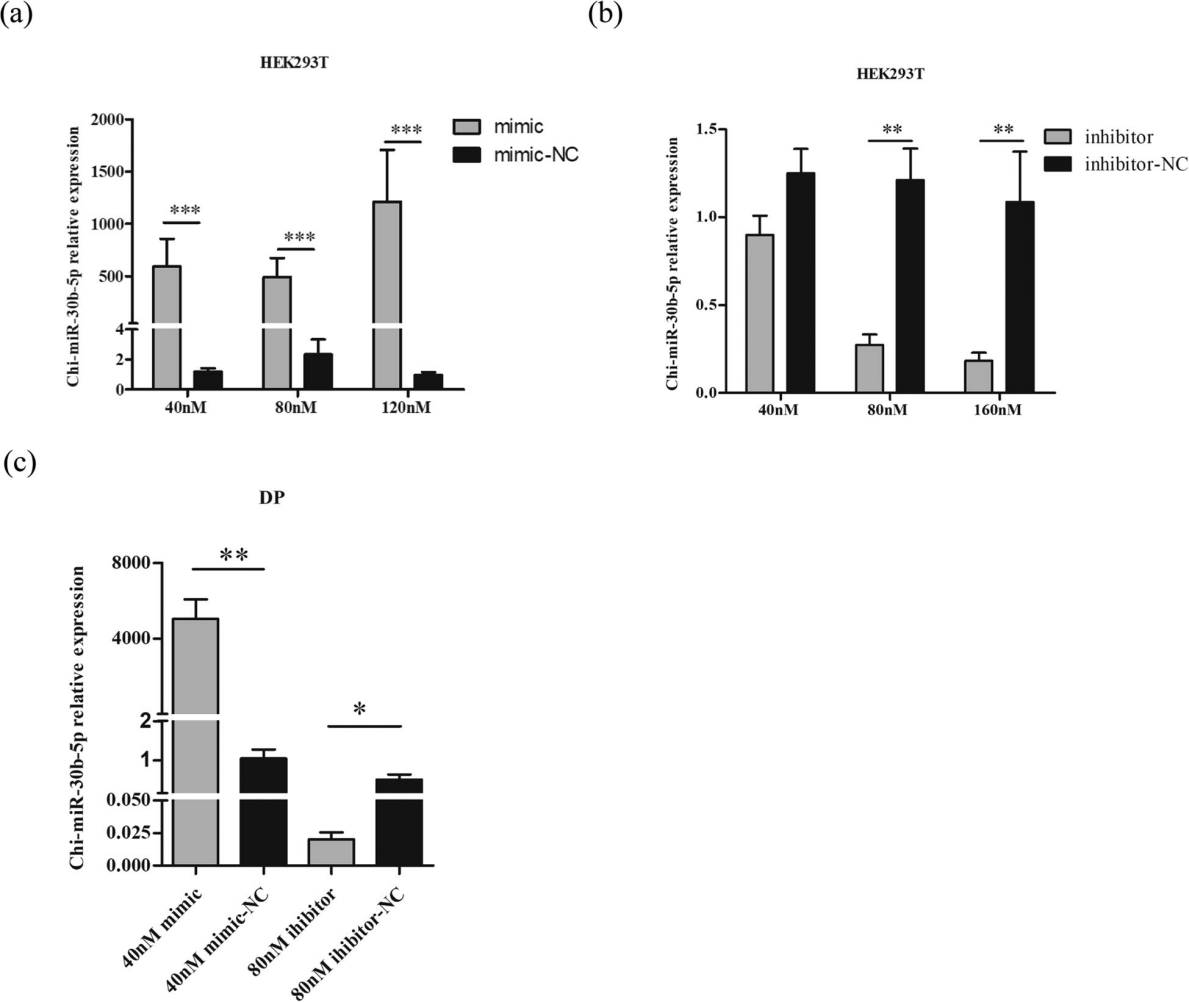
RT-qPCR of chi-miR-30b-p expression after transfection with mimic and inhibitor. (a) Transfection with 40 nM,80 nM and 120 nM chi-miR-30b-5p mimic in HEK293T cells for 48 h. (b) Transfection with 40 nM, 80 nM and 160 nM chi-miR-30b-5p inhibitor in HEK293T cells for 48 h. (c) Transfection with 40 nM mimic and 80 nM inhibitor in DP cells for 48 h. The expression of chi-miR-30b-p was normalized to U6. Values are mean ± SEM for 3 biological replicates, *P < 0.05, **P < 0.01, ***P < 0.001

In order to further identify the miRNAs related with HF cycling, the differentially expressed miRNAs in our study were compared with Yuan et al’s results in the anagen and telogen [21]. Six miRNAs (chi-miR-150, chi-miR-151-5p, chi-miR-196a, chi-miR-196b, chi-miR-30b-5p, chi-miR-9-5p) were identified (Fig. 3a). Chi-miR-30b-5p was selected for further study combining with its abundant expression from our sequencing data and its function in tumor cell proliferation. The RT-qPCR result showed that chi-miR-30b-5p was significantly lower in the anagen than that in the telogen, which was consistent with the sequencing result (Fig. 3b). To evaluate the function of chi-miR-30b-5p, its mimic and inhibitor were transfected into HEK293T cells, respectively (Fig. 4a and b). The transfection efficiency was detected by RT-qPCR, and the results revealed that the supplementation of 40, 80 and 120 nM chi-miR-30b-5p mimic all significantly increased the expression level of chi-miR-30b-5p compared with the mimic-NC group, respectively (Fig. 4a). In contrast, the supplementation of 80 and 160 nM inhibitors resulted in significant decrease of chi-miR-30b-5p expression as compared to inhibitor-NC group (Fig. 4b). Therefore, 40 nM mimic and 80 nM inhibitor were transfected into DP cells for the subsequent assays, respectively. The RT-qPCR analysis revealed that they both could significantly affect the expression of chi-miR-30b-5p in DP cells (Fig. 4c).

**Fig. 5:**
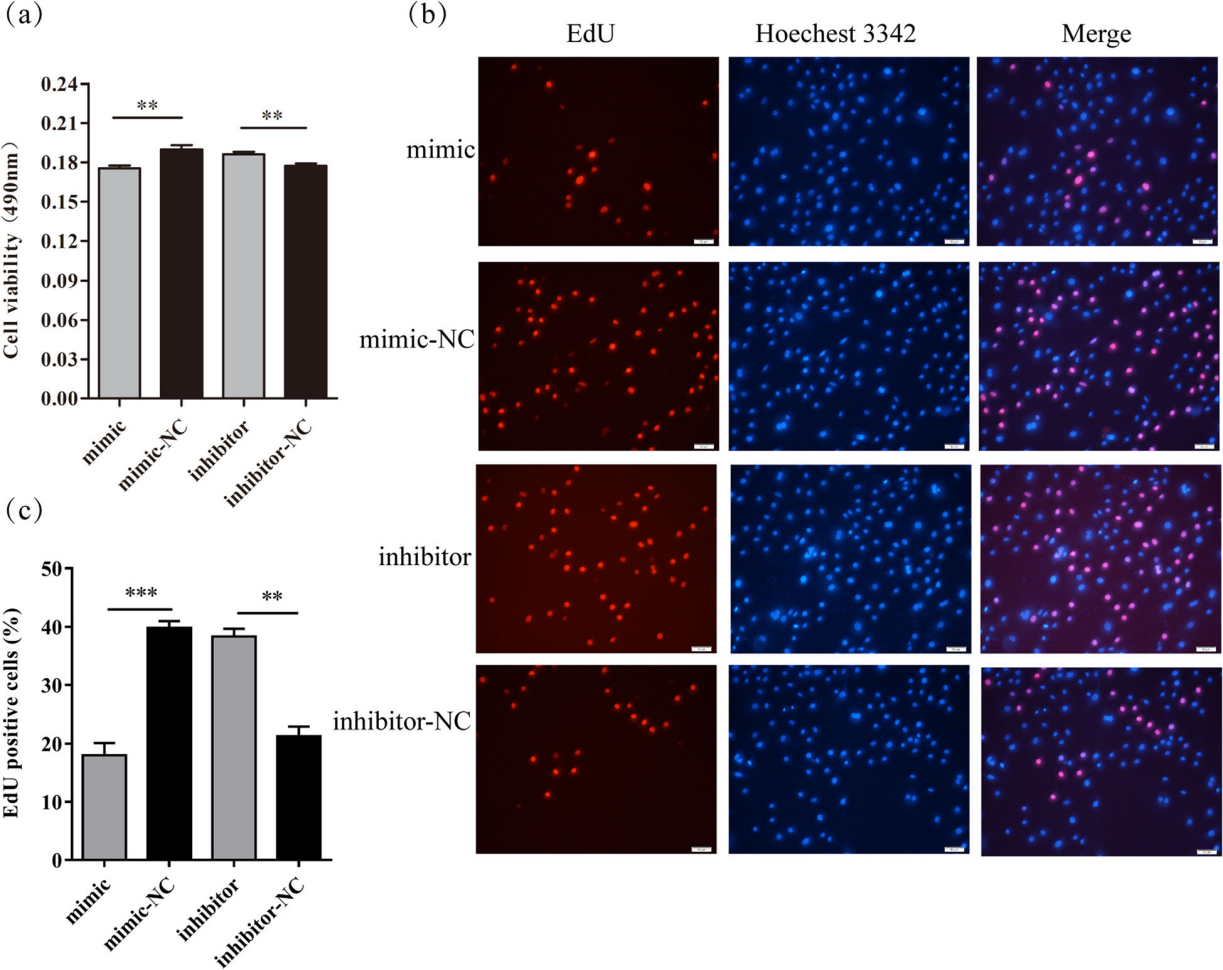
Effect of chi-miR-30b-5p on DP cells viability and proliferation. (a) DP cells were transfected with chi-miR-30b-5p mimic and inhibitor respectively. Cell viability was determined by MTT assay. (b) DP cells were transfected with chi-miR-30b-5p mimic and inhibitor, respectively. Cells proliferation was determined by EdU assay, the scale bar stands for 50 μ0. (c) Analysis results of EdU positive cells. The data were presented as mean ± SEM for 3 biological replicates, **P < 0.01, ***P < 0.001

Further, MTT and EdU assays were performed to explore the cell proliferation after the transfection of 24 h with chi-miR-30b-5p mimic or inhibitor. Cell viability was significantly reduced in DP cells with chi-miR-30b-5p mimic compared with the control group (*P* < 0.01), whereas cell viability was significantly increased with the supplementation of chi-miR-30b-5p inhibitor compared to its control (*P* < 0.01) (Fig. 5a). Similarly, the cultured DP cells had significantly less mitotic activity (low percent of EdU positive cells) with chi-miR-30b-5p overexpression and more mitotic activity (high percent of EdU positive cells) with chi-miR-30b-5p suppression (Fig. 5b and c). Combined with the MTT result, it could be confirmed that chi-miR-30b-5p inhibited the proliferation of DP cells.

### Chi-miR-30b-5p directly targets the 3’UTR of CaMKIIδ gene

**Fig. 6:**
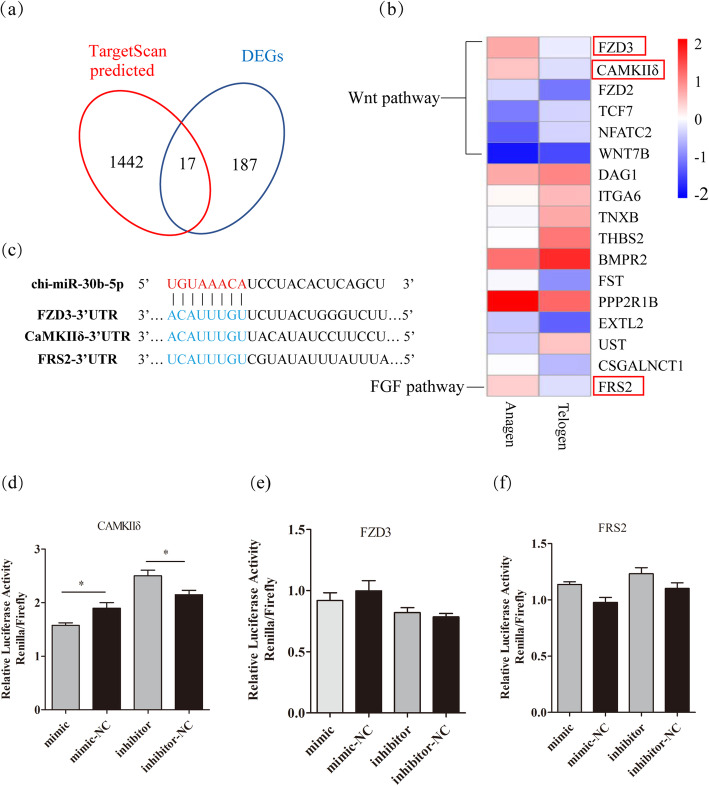
Chi-miR-30b-5p directly targets *CaMKIIδ* gene. (a) A Venn diagram depicting the overlap of the results of chi-miR-30b-5p targets predicted by Targetscan databases with different expressed genes (*p*-value < 0.005, were included in the analysis) related with HF development in anagen and telogen. (b) 17 DEGs were shown in Heat Map. (c) Sequence of chi-miR-30b-5p and its predicted binding site in FZD3–3’UTR, CaMKIIδ-3’UTR and FRS2–3’UTR. (d) Dual luciferase assay to detect the interaction between chi-miR-30b-5p and CaMKIIδ-3’UTR, after co-transfecting the corresponding report vectors with miR-30b-5p mimic or inhibitor in HEK293T. (e) The dual luciferase assay with chi-miR-30b-5p and FZD3–3’UTR. (f) The dual luciferase assay with chi-miR-30b-5p and FRS2–3’UTR. The data were presented as mean ± SEM for 3 biological replicates, *P < 0.05

To identify the direct targets of chi-miR-30b-5p in DP cells, the DEGs (*p* value < 0.05) associated with HF development in the anagen and telogen were conjointly analyzed with the potential targets of chi-miR-30b-5p predicted by TargetScan (Fig. 6a). Three potential targets of chi-miR-30b-5p (Fig. 6b), *FZD3*, *CaMKIIδ*, and *FRS2* genes expressed significantly higher in the anagen than that in the telogen from RNA-seq profile [22]. Dual-luciferase assay was performed to validate and explore whether chi-miR-30b-5p could bind to the 3’UTRs of *FZD3*, *CaMKIIδ* and *FRS2* or not (Fig. 6c). The renilla luciferase activity was significantly reduced after the co-transfection of *CaMKIIδ* 3’UTR psi-check2 reporter with chi-miR-30b-5p mimic compared with the corresponding control, whereas it was significantly increased with the transfection of inhibitor (Fig. 6d). However, *FZD3* and *FRS2* genes had no significant changes (Fig. 6e and f). The results indicated that *CaMKIIδ* was a target gene of chi-miR-30b-5p other than *FZD3* and *FRS2.*

### Chi-miR-30b-5p inhibits the expression of CaMKIIδ

**Fig. 7:**
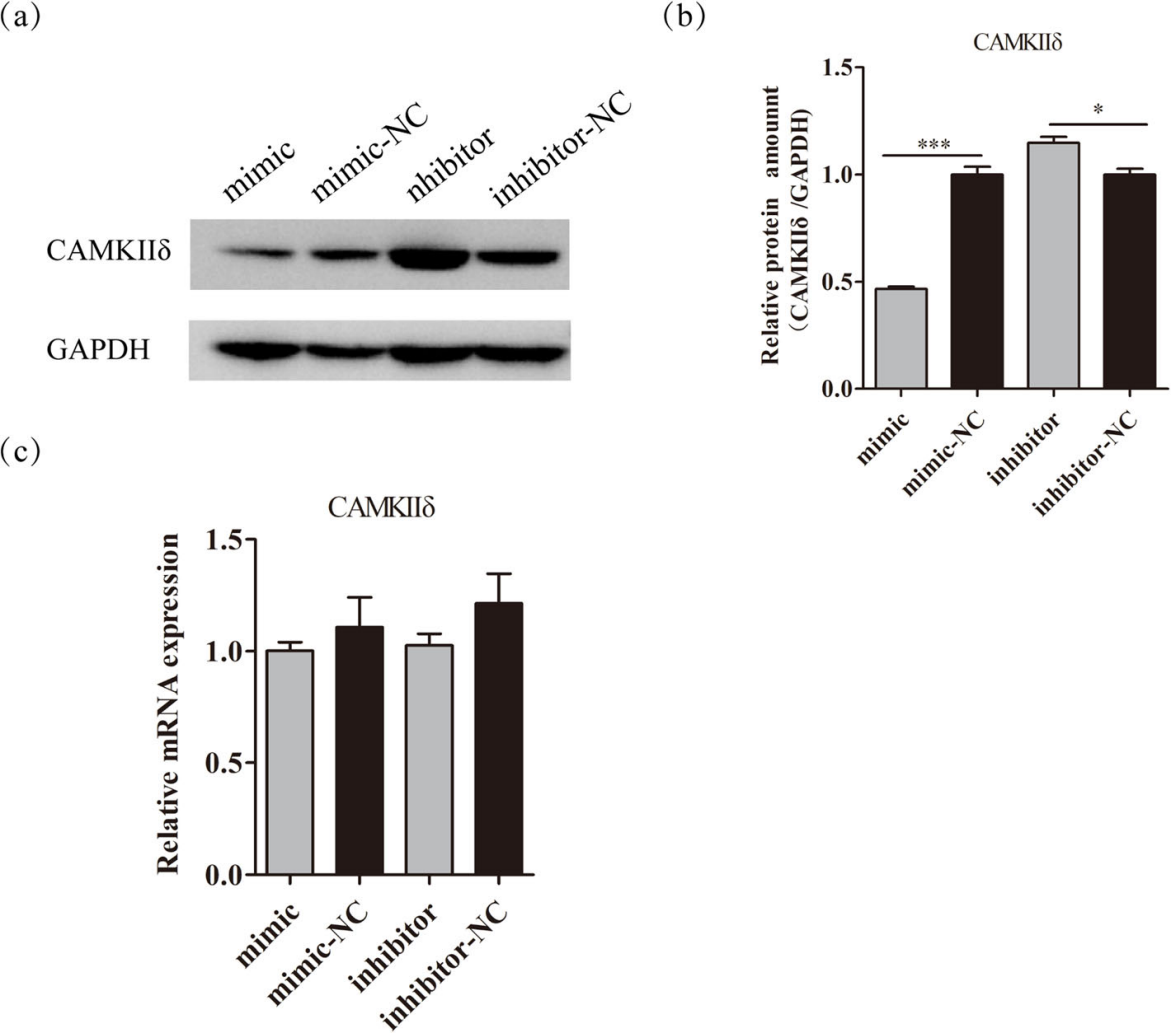
Chi-miR-30b-5p inhibits the expression of CaMKIIδ at protein level. (a) CaMKIIδ protein expression was detected by western blot. (b) Gray value analysis normalized to GAPDH. (c) The expression of *CaMKIIδ* gene was detected by RT-qPCR normalized to *β-actin*. The data were presented as mean ± SEM for 3 biological replicates, *P < 0.05, ***P < 0.001

To confirm the effect of chi-miR-30b-5p on *CaMKIIδ* translation in DP cells, the CaMKIIδ protein levels were examined with the transfection of mimic and inhibitor by Western blot. The result indicated that the overexpression of chi-miR-30b-5p suppressed the expression of CaMKIIδ, whereas the inhibition of chi-miR -30b-5p promoted CaMKIIδ protein expression (Fig. 7a and b). However, the mRNA expression level of *CaMKIIδ* was not significantly changed with the overexpression or inhibition of chi-miR-30b-5p in DP cells (Fig. 7c), suggesting that chi-miR-30b-5p could inhibit the translation of *CaMKIIδ* gene. Thus, we concluded that chi-miR-30b-5p directly targeted the 3’UTR of CaMKIIδ to inhibit its translation in DP cells.

## Discussion

MiRNA can play an important role in developmental timing, metabolism, occurrence of disease, and cell proliferation by gene regulation in animals and plants [23–26]. High-throughput sequencing technology has been widely used in identifying the differential expressions of miRNAs in various species [27–29]. Cashmere is produced by the secondary HF, and its growth has obvious periodic changes: anagen (growth), catagen (cessation of growth) and telogen (resting) [30]. One of the most important events in HF growth process is the transition from telogen to anagen phase, which called hair regrowth. It has known that miR-214, miR-21, miR-24 and miR-196a play important roles in human or mouse skin and HF development [14–16]. In current study, a total of 58 annotated miRNAs and 3 novel significantly differentially expressed miRNAs, including chi-miR-214-3p and chi-miR-196a, were identified between the anagen and telogen through miRNA-seq, indicating that these two miRNAs may participate in the regulation of follicular transition. The target genes of differentially expressed miRNAs were significantly enriched in Wnt, VEGF, ECM-receptor interaction, FGF and BMP Signaling pathways. The result was consistent with the studies in humans and mice hair follicle development [3, 31–33].

Several previous researches had reported the discovery and identification of miRNAs in goat skin by sequencing [21, 34]. Corresponding with Yuan et al’s result [21], chi-miR-30b-5p expressed significantly lower in the anagen than that in the telogen. Therefore, chi-miR-30b-5p was selected for further investigation. Zhu et al. found that miR-30b-5p could suppress cell proliferation and promote cell apoptosis in gastric cancer [19]. DP cells, as an important cell model playing a vital role in hair formation [6, 35, 36], were used to investigate the function of chi-miR-30b-5p. In this study, we found that chi-miR-30b-5p inhibited DP cells proliferation through MTT and EdU assays. The cell viability and mitotic activity of DP cells were significantly reduced with the overexpression of chi-miR-30b-5p.

Primary function of miRNAs is to reduce mRNA decay or inhibit translation of their target genes [37]. Reports show that miR-30b-5p inhibits human colorectal cancer by targeting *KRAS*, *PIK3CD* and *BCL2* [20] and modulates glioma cell proliferation by directly targeting *MTDH* [24]. In order to illustrate the molecular mechanism of chi-miR-30b-5p on DP cells proliferation, the potential target genes were predicted and screened by TargetScan combining the RNA-seq data from skin tissues at the anagen and telogen of Shanbei white cashmere goat [22]. *CaMKIIδ* was selected as a target gene of chi-miR-30b-5p. CaMKIIδ is one of the four isoforms of CaMKII (Ca2+/calmodulin-dependent protein kinase II), a serine/threonine protein kinase with a broad spectrum of substrates [38–40]. CaMKII is an important member of Wnt/Ca^2+^ pathway which activates Ca^2+^/calmodulin-dependent protein kinase II (CaMKII), and thus multiple downstream signal pathways [41]. The dual-luciferase assay demonstrated that chi-miR-30b-5p directly targeted the 3’UTR of *CaMKIIδ* gene. And the overexpression of miR-30b-5p significantly decreased the CaMKIIδ expression at protein level, but not at mRNA level. Obviously, our data indicated that miR-30b-5p could inhibit the translation of *CaMKIIδ* gene. A previous study revealed that miR-30b-5p was down-regulated in cardiac hypertrophy, and the restoration of its function inhibited the expression of *CaMKIIδ*, thereby preventing cellular hypertrophy. Their findings suggested that miR-30b-5p might function as a hypertrophic suppressor [42]. Consistent with their results, we found that chi-miR-30b-5p could inhibit cell proliferation by suppressing the expression of CaMKIIδ at protein level in DP cells.

## Conclusions

**Fig. 8:**
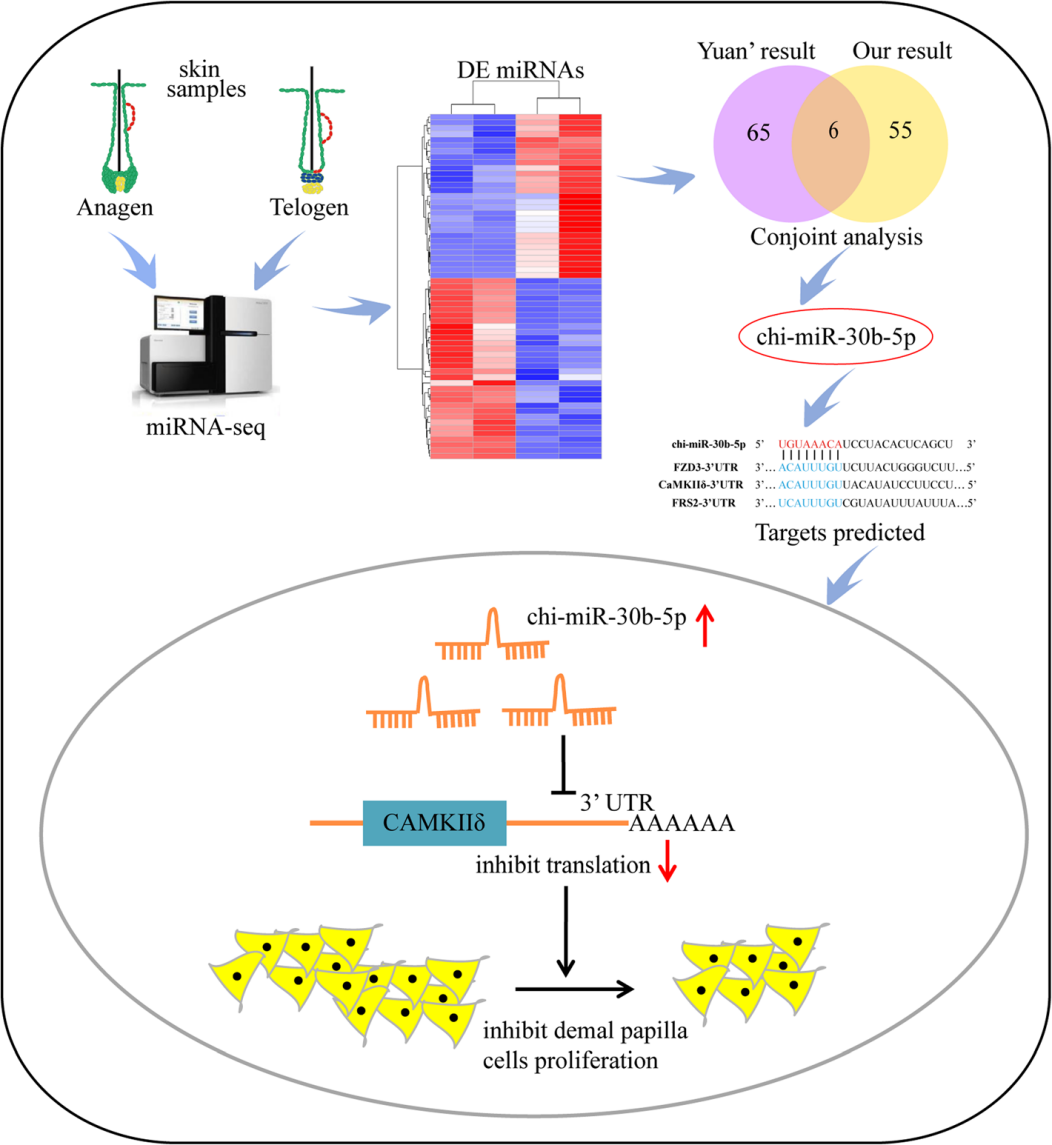
Working model of miRNAs filtration and regulatory network diagram view of chi-miR-30b-5p on DP cell proliferation

In summary, we identified 61 differentially expressed miRNAs in anagen and telogen. Among them, we proved that chi-miR-30b-5p could inhibit DP cells proliferation by inhibiting *CaMKIIδ* translation (Fig. 8). Our study provides an additional insight into understanding the regulatory mechanism of hair follicle growth.

## Methods

### Tissues collected, RNA isolation and sequencing

Six Shanbei White cashmere goats were from Shanbei Cashmere Goats Engineering Technology Research Center (Shaanxi, China) and randomly separated into two groups. The skin tissues were collected at cashmere anagen and telogen, respectively. Acquisition of cashmere goat skin samples had been described in our previous article [22]. After sample collection, all the animals were raised normally and recovered in two weeks with appropriate care. Total RNA was extracted from the obtained skin tissues using Trizol reagent (Invitrogen, USA). The concentration and quality of total RNA were detected using an Agilent Bioanalyzer 2100 and stored at − 80 °C. For miRNA-Seq, four miRNA pool libraries were constructed from anagen (*n* = 2) and telogen (n = 2). Total RNA (3 μg) from three individuals at the same stage in each pool was adopted as input material for the miRNA library. An Illumina Hiseq 2500 platform was used in the present study and 50 bp single-end reads were generated.

### Sequence analysis

The acquisition of clean data (clean reads), annotated miRNA and novel miRNA prediction was defined in previous study [22]. The clean reads with Q20, Q30 > 99% and the length between 18 ~ 35 nt were subjected to downstream analysis. The genome mapped software Bowtie (v-0.12.9) was used to alignment with the filtered sRNA clean reads [43].

### Differential expression of miRNAs

Transcript per million (TPM): Normalization formula: Normalized expression = (actual miRNA count/total count of clean reads) *1000000 were used for assessing miRNA expression level analysis [44]. Differential miRNA expression analysis between the anagen and telogen was executed using the DESeq2 R package. Hochberg and Benjamini method were used to standardize the *p*-values [45]. Clustering analysis of differentially expressed miRNAs was achieved utilizing pheatmap R package.

### MiRNA targets prediction and KEGG pathway analyses

MiRanda (http://www.microrna.org/microrna/home.do) and TargetScan (http://www.targetscan.org/vert_71/) were used to predict the potential targets of miRNAs. The miRNA-mRNA interaction network was established and visually presented using Cytoscape software (v3.6.1). Then the predicted targets were conjointly analyzed with the DEGs (q value ≤0.05) in anagen and telogen. KOBAS 3.0 (http://kobas.cbi.pku.edu.cn/index.php) was used to investigate the statistical enrichment of candidate targets in KEGG pathways.

### Real-time quantitative PCR (RT-qPCR)

Extracted RNA from cells or tissues was converted to cDNA using the PrimeScript™ RT regent Kit with gDNA Eraser (Takara, Dalian, China). RT-qPCR was executed on a Bio-Rad CFX96 Touch™ Real Time PCR Detection System (Bio-Rad, USA) with TB Green™ *Premix Ex Taq*TM II (Takara, Dalian, China). RT-qPCR procedure was as follows: 95 °C for 1 min, then following 40 cycles of 95 °C for 10 s and appropriate annealing temperatures for 30 s. RT-qPCR primers were listed in Additional file 8 and designed by Primer Premier 6.

### Vector construction

*FZD3*, *CaMKIIδ* gene involving Wnt signal pathway and *FRS2* gene in FGF signal pathway were considered as the potential targets of chi-miR-30b-5p by TargetScan software. The FZD3–3’UTR, CaMKIIδ-3’UTR and FRS2–3’UTR sequences including chi-miR-30b-5p binding site were amplified by PCR, then the amplified fragments and psi-CHECK-2 dual-luciferase reporter vector (Promega, Madison, WI, USA) were digested by *Xho* I/*Bsa* I and *Not* I enzymes (NEB, New England) and subsequently ligated by T4 DNA ligase (NEB, New England). The constructed vectors were further verified by sequencing. The PCR primer information was listed in Additional file 8.

### Cell culture and transfection

HEK293T cell line (GNHu17, Cell Bank of the Chinese Academy of Sciences, Shanghai, China) was cultured in high-glucose Dulbecco’s modified Eagle’s medium (DMEM; Hyclone, USA) supplemented with 1% double antibiotics (Penicillin and Streptomycin) (GM) and 10% fetal bovine serum (FBS; Hyclone, USA) in a 37 °C cell incubator with 5% CO_2_. The DP cells were isolated from the secondary HFs of cashmere goat and cultured in DMEM/F12 media as previous described [4]. When the cells reached 80% confluence were transfected with chi-miR-30b-5p mimic, chi-miR-30b-5p inhibitor at an optimal concentration using Lipofectamine 2000 transfection reagent ((Invitrogen, Carlsbad, CA) and Opti-MEM (Gibco). The transfection efficiency was identified by RT-qPCR.

### Cell proliferation assays

#### MTT assay

96-well plate was used to seed single cell pellets with 1 × 10^4^ cells per well. The mimic (40 nM) and inhibitor (80 nM) of chi-miR-30b-5p were transfected into DP cells using Lipofectamine 2000. After the transfection of 24 h, the cultured medium was aspirated, then added 50 μL of MTT (3-(4,5-dimethylthiazol-2-yl)-2,5-diphenyltetrazolium bromide, 0.5 mg/mL, Sigma, St. Louis, MO) to each well, and the plates were further incubated for 4 h in a 37 °C incubator in the dark. Then, 150 μL of dimethyl sulfoxide (DMSO) (D8371, Solarbio) was added to each well to dissolve the formed formazan crystals. Cell viability was measured using optical density at 490 nm (OD490) absorbance value ratios using a SynergyH1 multi-detector microplate reader (BioTek, Winooski, VT).

#### EdU proliferation assay

DP cell proliferation was also evaluated using Cell-Light EdU DNA cell proliferation kit (RiboBio, Guangzhou, China). The DP cells were cultured for 24 h in a 96-well plate, then incubated with EdU medium for 2 h and the EdU positive cells were detected following the manufacturer’s protocol.

#### Dual-luciferase activity assay

The chi-miR-30b-5p mimic (40 nM), inhibitor (80 nM), and their corresponding negative controls were cotransfected into HEK293T cells with psiCHECK-2-FZD3–3’UTR, psiCHECK-2-CaMKIIδ-3’UTR, or psiCHECK-2-FRS2–3’UTR vector when the cells were about 80% confluence in 24-well plates by Lipofectamine 2000, respectively. The transfection reagent was changed with fresh growth medium (DMEM with 1% double antibiotics, 10% FBS) after the transfection of 6 ~ 8 h. Then the cells were rinsed with PBS one time and harvested following the manufacturer’s protocol. The luciferase activities were measured by dual luciferase reporter kit (Promega) and the Renilla Luciferase activity was normalized against Firefly Luciferase activity. The transfections were performed in triplicate.

### Western blot

The process of total proteins extraction and protocol of western blot were described in previous study [46]. The antibody information was as follows: anti-CaMKIIδ (ab181052; Abcam, England), anti-GAPDH (60004–1-Ig; proteintech, USA), secondary antibodies of anti-immune rabbit IgG-HRP (Zhongshan-Bio, Beijing, China) for CaMKIIδ and anti-immune-mouse IgG-HRP (Zhongshan-Bio, Beijing, China) for GAPDH. All antibodies were diluted into 1:1000.

### Statistical analysis

The fold-change of mRNA or miRNA expression was analyzed using the 2^-△△CT^ method. *β-actin* or *U6* was used as internal control to normalize the data. RT-qPCR data were presented as mean ± standard error of the mean (SEM). Histograms in this research were analyzed by GraphPad Prism 7.0 software (GraphPad Software, San Diego, CA, USA) with t-test. Statistical significance was considered as *P < 0.05, **P < 0.01, and ***P < 0.001. Image J was handled for gray value analysis of gels image.

## Supplementary information

**Additional file 1.** List of sRNA quantity and type after length screening (18-35 nt) and mapped to goat genome.

**Additional file 2.** The information of mapped sRNA.

**Additional file 3.** The information of mapped annotated miRNA.

**Additional file 4.** The information of novel miRNAs.

**Additional file 5.** The expression levels of annotated and novel miRNAs (normalized by TPM).

**Additional file 6.** The differentially expressed miRNAs of annotated and novel miRNAs (analyzed by DESeq2).

**Additional file 7.** The information of KEGG.

**Additional file 8.** The information of primers.

## Data Availability

The miRNA-seq datasets supporting the conclusions of this paper are available in the NCBI SRA repository (https://www.ncbi.nlm.nih.gov/sra/). BioProject accession: PRJNA572591. BioSamples: SAMN12796315, SAMN12796316, SAMN12796317, SAMN12796318. The lncRNA-seq datasets supporting the DEGs used in this study are available in the NBCI SRA repository. BioProject accession: PRJNA477237. BioSamples: SAMN09464004, SAMN09464005, SAMN09464006, SAMN09464007, SAMN09464008, SAMN09464009. The miRNA-seq datasets supporting the differentially expressed miRNAs in Yuan et al’s results used the current research are available in Gene Expression Omnibus (GEO) (https://www.ncbi.nlm.nih.gov/gds/) with the accession number GSE47742.

## Abbreviations

DP: Dermal papilla
miRNA: microRNA
miRNA-Seq: miRNA sequencing
HF: Hair follicle
DEGs: Differentially expressed genes
KEGG: Kyoto encyclopedia of genes and genomes
RT-qPCR: Real-time quantitative PCR
sRNA: Small RNA
TPM: Transcript per million
